# Clinical attachment loss and molecular profile of inflamed sites before treatment

**DOI:** 10.1590/1678-7757-2018-0671

**Published:** 2019-08-30

**Authors:** Cristine D'Almeida Borges, Milla Sprone Ricoldi, Michel Reis Messora, Daniela Bazan Palioto, Sérgio Luís Scombatti de Souza, Arthur Belém Novaes, Mario Taba

**Affiliations:** 1 Universidade de São Paulo Universidade de São Paulo Faculdade de Odontologia de Ribeirão Preto Departamento de Cirurgia e Traumatologia Bucomaxilofacial e Periodontia São Paulo Brasil Universidade de São Paulo, Faculdade de Odontologia de Ribeirão Preto, Departamento de Cirurgia e Traumatologia Bucomaxilofacial e Periodontia, Ribeirão Preto, São Paulo, Brasil.

**Keywords:** Periodontal attachment loss, Biological markers, Gingival crevicular fluid, Biopsy, Gene expression

## Abstract

**Objective:**

To monitor early periodontal disease progression and to investigate clinical and molecular profile of inflamed sites by means of crevicular fluid and gingival biopsy analysis.

**Methodology:**

Eighty-one samples of twenty-seven periodontitis subjects and periodontally healthy individuals were collected for the study. Measurements of clinical parameters were recorded at day −15, baseline and 2 months after basic periodontal treatment aiming at monitoring early variations ofthe clinical attachment level. Saliva, crevicular fluid and gingival biopsies were harvested from clinically inflamed and non-inflamed sites from periodontal patients and from control sites of healthy patients for the assessment of IL-10, MMP-8, VEGF, RANKL, OPG and TGF-β1 protein and gene expression levels.

**Results:**

Baseline IL-10 protein levels from inflamed sites were higher in comparison to both non-inflamed and control sites (p<0.05). Higher expression of mRNA for IL-10, RANK-L, OPG, e TGF-β1 were also observed in inflamed sites at day −15 prior treatment (p<0.05). After the periodontal treatment and the resolution of inflammation, seventeen percent of evaluated sites still showed clinically detectable attachment loss without significant differences in the molecular profile.

**Conclusions:**

Clinical attachment loss is a negative event that may occur even after successful basic periodontal therapy, but it is small and limited to a small percentage of sites. Elevated inflammation markers of inflamed sites from disease patients reduced to the mean levels of those observed in healthy subjects after successful basic periodontal therapy. Significantly elevated both gene and protein levels of IL-10 in inflamed sites prior treatment confirms its modulatory role in the disease status.

## Introduction

Periodontal disease is a chronic microbial infection characterized by the inflammation of supportive tissues and alveolar bone loss. Particularly in chronic periodontitis, the presence of local irritants is compatible with the severity of the disease.^1^ Although bacteria are essential in the onset and maintenance of periodontitis, susceptibility and disease progression are determined by a complex interaction driven by the modulation of an immune-inflammatory host response.^2^^,^^3^ Locally, bacterial lipopolysaccharides induce inflammatory cells to release pro-inflammatory mediators that seem to act in the destruction of periodontal tissues.^3^ The presence of inflammatory cells and lymphocytes infiltration, chemotactic factors involved in recruiting these cells and cytokines involved in the pathogenesis and progression of the periodontal disease.^4^

The activation of a local immune response by T helper cells would determine the stability or progression of the periodontal disease. Th1 lymphocytes are characterized by the secretion of cytokines involved in eradicating intracellular pathogens, whereas Th2 cells are responsible for secreting cytokines involved in eliminating extracellular micro-organisms.^5^ Also, Th17 and T regulatory (Treg) cells are involved in disease progression. Th17 subset presents pro-inflammatory and pro-resorptive activities, especially for secretion of IL-17 and RANKL, both involved in the differentiation and activation of osteoclasts. On the other hand, Treg cells subset displays suppressor functions producing IL-10 and transforming growth factors (TGF-β_1_).^6^^,^^7^ In this context, IL-10 seems to have a modulatory role on inflamed and progressive sites.

Host modulatory effects of specific cytokines such as IL-10, IL-13, OPG and TGF-β_1_ are responsible for the selective recruitment of different cells, cytokines production and may determine the disease progression.^8^ These cytokines associated to host defense have been identified in saliva,^3^^,^^9^ blood,^10^^,^^11^ gingival crevicular fluid^8^^,^^11^ and gingival tissues.^12^^,^^13^ Elevated levels of these molecules may be related to periodontal disease condition, allowing identification and controlling patients with periodontal disease.^14^

Studies that aim to analyze cytokines host modulatory effect during disease progression seem to be promising in periodontal diagnostic.^15^ Therefore, in this study, we aimed to monitor early changes in attachment levels of progressive sites and investigate clinical and molecular features of progressive sites through saliva, gingival crevicular fluid and gingival tissue samples.

## Methodology

### Patient population

Twenty-seven participants were selected; amongst them eighteen presented periodontitis stage II grade B^16^ (periodontitis group) and nine were healthy (control group). *Post hoc* power analysis was made through G*Power 3.1.9.2 using mean and standard deviations of the total amount level of IL-10 in inflamed and control sites, and 99% of power was obtained in this study.

Participants were chosen from the dental clinics of the Ribeirão Preto School of Dentistry and were invited to take part in the study. All enrolled patients gave written consent on a form approved by the Ethics Committee Protocol of the Ribeirão Preto School of Dentistry -USP (approval number # 02841912.0.0000.5419). Participants underwent anamnesis, clinical and radiographic examination.

Included participants had at least 14 natural teeth and posterior occlusion stability. Participants in the chronic periodontitis group were at least 35 years old with 5 teeth presenting probing depth (PD) of ≥5 mm and clinical attachment loss of ≥3 mm.^17^ Participants in the control group had PD ≤3 mm in all teeth and plaque index and bleeding on probing values ≤20%. Participants presenting any disorder or ongoing medication usage were excluded. Also, they could not have received periodontal treatment in the past six months.

### Clinical parameters

**Figure 1:**
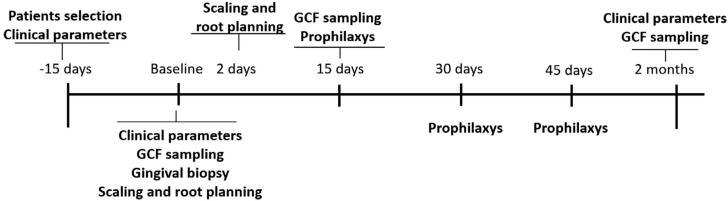
Timeline of the study

Clinical examinations and data collections were performed at day −15, baseline and two months after basic periodontal therapy. Figure 1 illustrates the timeline of the study. Probing pocket depth (PPD), relative clinical attachment level (rCAL) and bleeding on probing (BOP) were recorded at six sites *per* tooth (mesio-buccal, buccal, disto-buccal, mesio-lingual, lingual and disto-lingual) with the aid of a computerized periodontal probe (Florida Probe Corporation, Gainsville, FL, USA). The presence or absence of biofilm at four sites *per* tooth (plaque index -PI) were also recorded.^18^ It was also verified the furcation involvement with the aid of a manual periodontal probe (Hu-Friedy, Chicago, IL, USA).

After clinical examinations of day −15 and baseline, sites were categorized according to the presence or absence of inflammation: (i) inflamed sites (PD ≥ 5 mm and recurrent BOP after clinical exams at −15 days and baseline); (ii) non-inflamed sites (PD ≤4 mm without BOP after clinical exams at −15 days and baseline); (iii) and control sites (PD ≤3 mm without BOP after clinical exams at −15 days and baseline). For matching comparison purpose, inflamed sites and non-inflamed sites were from the same participant (periodontitis group) for gingival crevicular fluid and gingival biopsy analysis.

Scaling and root planning sessions were performed by the same operator in two to four sessions within 24- to 48-hour interval^19^ using hand instruments (Hu-Friedy, Chicago, IL, USA) and an ultrasonic (Dentsply, York, PA, USA) device. Oral hygiene was reviewed after a week and repeated 30 days after periodontal disinfection, followed by dental prophylaxis. After two months, a new periodontal examination was performed to evaluate PI, BOP, PD and rCAL using a computerized probe to detect progressive sites.

Progressive sites categorization was based on the tolerance method.^20^^,^^21^ In brief, progressive sites were those that presented clinical attachment loss of ≥1 mm after two months considering the average error of 0.3 mm of the electronic probe multiplied by 3.

Scaling and root planning sessions, clinical examinations and data collections were made by only one examiner, who is an experienced Periodontist (Borges, C.D.).

### Saliva collection and analysis

The patients were instructed not to drink or eat for at least 60 min before the saliva sample collection. Non-stimulated whole expectorated saliva was collected (~3 ml) from each subject into sterile tubes, according to the method described by Navazesh^22^ (1993), by one calibrated examiner the day after the initial diagnosis and on the day after post-therapy periodontal evaluation. Saliva samples were placed on ice immediately and aliquoted prior to freezing at −80°C.

The salivary inflammatory protein levels were identified simultaneously using Multiplex Cytokine Profiling Assay in the Luminex platform (Luminex Corporation, Austin, TX, USA). The following proteins were analyzed: IL-10, MMP-8, VEGF, RANKL, OPG and TGF-β_1_. The assay was performed according to the manufacturer's protocol. Briefly, ten microliters of the diluted sample (proteins) were added to a 50 µl cocktail of capture beads and an antibody detector, and the mixture was incubated for 4 hours at room temperature. Excess unbound antibody detector was washed off and flow cytometric analysis were performed using the appropriate CMA analysis software.

### Gingival crevicular fluid sampling and analysis

Gingival crevicular fluid samples were collected at baseline, 15 days and 2 months after therapy. In periodontitis group patients, gingival crevicular fluid samples were collected from three inflamed sites and one non-inflamed site. In control patients, fluid samples were collected from one control site. First, the supragengival plaque was removed, sites were isolated with cotton rolls and gently air dried. Fluid samples were collected with sterile Periopaper strips (Oraflow Inc., Planviwe, NT, USA) that were inserted into the gingival crevice until mild resistance was felt and left in place for 30 seconds. After gingival crevicular fluid collection, strips were placed in Eppendorf vials and immediately frozen at −80°C until use.

Gingival crevicular fluid samples were placed into 60 µl of sodium phosphate buffer (Life Technologies, Carlsbad, California, USA) and 0.01 ml of Tween^®^ 20 (USB Corporation, Cleveland, USA). Protein levels of IL-10 and VEGF were identified simultaneously using multiplex cytokine profiling assay (Luminex Corporation, Austin, TX, USA). MMP-8 levels were analyzed by ELISA and carried out according to the manufacturer's instructions.

### Gingival biopsy

For collecting gingival tissue samples (containing both epithelial and connective tissues), all patients received local anesthesia. In periodontitis group patients, samples were harvested from one inflamed and one non-inflamed site. In Control patients, samples were removed from one control site. The gingival biopsies were harvested from the same site that had the gingival crevicular fluid collected. Two incisions were made for samples collection. First, the initial incision was made 1.5 mm away from the tooth with a scalpel, until bone crest. Then, an intracrevicular incision was made for gingival tissue removal that consists of periodontal pocket/gingival sulcus wall. Incisions were made around the selected sites, not around the tooth. In patients from periodontitis group, samples were removed during periodontal treatment, before scaling and root planning. In patients from control group, samples were removed during surgical procedures as root coverage. These samples were immediately flash frozen in liquid nitrogen then preserved under −80°C for posterior RNA extraction and gene expression analysis of IL-10, MMP-8, VEGF, RANKL, OPG e TGF-β_1_.

### RNA extraction and Real-time PCR

Total RNA from biopsies was extracted using the Trizol reagent (Invitrogen, Milan, Lombardy, Italy) method. The aqueous phase was transferred to a new tube, to which 0.25 ml of 95% ethanol (Sigma, St Louis, MO, USA) was added. The suspension was transferred to the spin basket assembly of the kit (Promega, Madison, WI, EUA) and centrifuged at 10,500 rpm for 1 min at 4°C. From 1 µg of total RNA, a strand of complementary DNA (cDNA) was synthesized through a reverse transcription reaction (SABioscience, Frederick, MD, USA).

Reactions were carried out in triplicate for each sample (inflamed sites, non-inflamed sites and control sites). The reactions were performed on a real-time thermocycler (Life Applied Biosystems, Carlsbad, California, USA), according to the directions supplied by the manufacturer. Following sample amplification and calculations, the expression levels were determined.

### Statistical analysis

Data were grouped by average and their respective standard deviation. Specific sites and individuals were considered for parametric or non-parametric statistical analysis when appropriated after Lilliefors normality test.

### Clinical parameters

For intra-group comparison, before and after treatment, Wilcoxon test or t test was applied. For intergroup comparison, Mann-Whitney test or t test was applied. A significance level of 5% was adopted for all statistical analyses (P<0.05).

### Gingival crevicular fluid proteins

For intra- and intergroup comparison, at baseline, 15 days and 2 months, Kruskal-Wallis test or ANOVA was applied.

### Real Time PCR arrays

Differential expression calculation was done by a specific software for data analysis (SABiosciences, Frederick, MD, USA). Relative gene expression normalization and quantification were performed by 2-ΔΔCT method.^23^ This software also performed pair-wise comparisons between groups of experimental replicates and defined fold-change and statistical significant thresholds. Therefore, data were presented as a difference (fold regulation) in gene expression, which would be normalized by the geometric mean value of actin-beta (ACTB). Significance level was set at *p*<0.05.

## Results

### Clinical findings

The subjects’ demographic data are displayed in Table 1. There was a higher prevalence of women and Caucasians in our sample. At baseline, periodontitis and control groups had different mean values of clinical parameters (Table 1). After basic periodontal therapy, periodontitis group showed a significant improvement in the clinical parameters (*p*<0.05).

**Table 1:** Demographic and clinical data from Control and CP groups.

	Control (n = 9)	Periodontitis (n = 18)	*P Value
**Age (years; mean ± SD)**	33.2 ± 7.82	48.1 ± 7.82	p = 0.001¥
**Female (%)**	66,70%	72,20%	_
**Caucasian (%)**	100%	83,30%	_
**Non-Caucasian (%)**	0%	16,70%	_
**N. teeth**	27.4 ± 4.2	23.7 ± 2.6	0,007
**PPD (mm)**			
initial	2.2 ± 0.1	3.1 ± 0.6	p < 0.0001**
2 months	2.1 ± 0.1	2.4 ± 0.3	p = 0.0035**
*p* value	NS*	< 0.0001**	-
Delta (Δ)	0.1 ± 0.2	0.7 ± 0.4	p < 0.0001**
**rCAL (mm)**			
initial	8.3 ± 1.2	10.4 ± 1.2	p = 0.004¥
2 months	8.0 ± 1.0	9.5 ± 0.9	p = 0.0007¥
*p* value	NS**	0.0002*	-
Delta (Δ)	0.3 ± 0.3	0.9 ± 0.5	p = 0.0012**
**PI (%)**			
initial	11.1 ± 6.3	68.9 ± 21.5	p < 0.0001**
2 months	10.6 ± 6.0	31.8 ± 22.7	p = 0.0007¥
*p* value	NS**	< 0.0001**	-
Delta (Δ)	0.5 ± 4.8	37.1 ± 25	p < 0.0001**
**BOP (%)**			
initial	16.7 ± 10.3	49.3 ± 12.8	p < 0.0001¥
2 months	13.7 ± 7.7	27.2 ± 7.3	p = 0.0001**
*p* value	NS*	< 0.0001**	-
Delta (Δ)	3.1 ± 7.2	22.1 ± 13.3	p = 0.0005**

NS - non significant (p>0.05). Mean ± standard deviation.

* Wilcoxon test for intragroup comparisons;

** t test for intragroup comparisons and between two groups;

¥ Mann-Whitney test for comparisons between two groups at baseline and at 2-month evaluation.

PD: Probing depth; rCAL: Relative attachment level; PI: Plaque index; BOP: Bleeding on probe

Significant differences between inflamed and non-inflamed sites for PD, rCAL and BOP, and between inflamed and control sites for PD and PI (p<0.05) (Table 2) were also observed. 2,436 sites from periodontitis group were analyzed and after periodontal therapy, 17% of total sites showed progressive clinical attachment loss (p<0.05).

**Table 2:** Mean difference after periodontal therapy of inflamed, non-inflamed and control sites.

	PD (mm)	rCAL (mm)	PI (%)	BOP (%)
Inflamed sites	1.93 ± 0.72	1.3 ± 0.82	46.3 ± 41.44	38.89 ± 32.84
Non-inflamed sites	0.6 ± 0.7^a^	0.1 ± 1.0^a^	22.2 ± 73.2	- 22.2 ± 42.8
Control sites	0.2 ± 0.4^a^	0.4 ± 0.7	-11.1 ± 33.3^a^	0.0^a^

Mean ± standard deviation. Kruskal-Wallis test for comparison between inflammation, non-inflammation and control sites.

a significantly lower than inflammation sites (p<0.05)

Comparisons of clinical measurements between −15 days and baseline, without any interventional therapy, showed difference in PD (5.6±0.85 and 5.9±1.30, respectively) in inflamed sites, but not significant (p=0.37). For non-inflamed sites (2.7±0.6 and 2.5±0.9, respectively), difference was also not significant (p=0.39).

### Salivary proteins

In the baseline, higher expression of RANK-L in periodontitis group 2.99 pg/mL in comparison to control group 1.2 pg/mL (p=0.0313) was observed. OPG protein expression was higher in periodontitis group before therapy. After 2 months, a 40% reduction was observed (p=0.0002).

### Gingival crevicular fluid proteins

**Figure 2:**
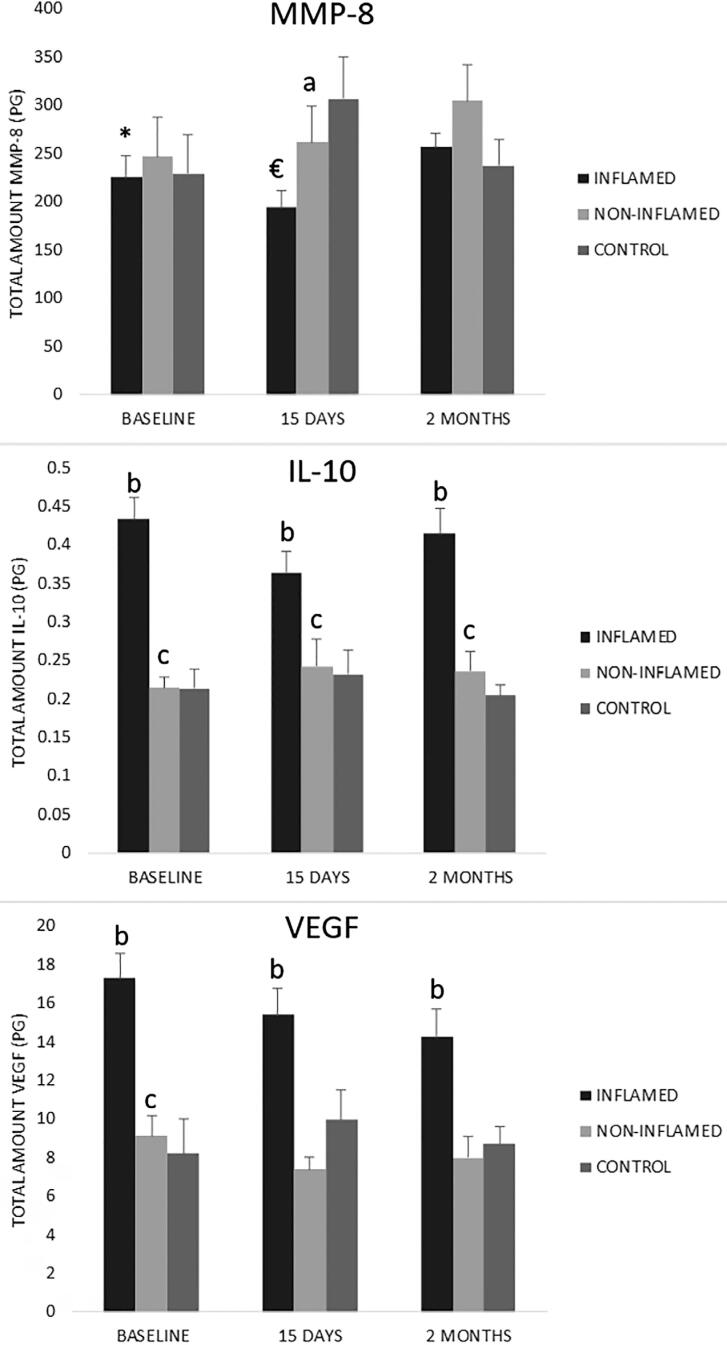
Total amount of VEGF, IL-10 and MMP-8 in gingival crevicular fluid of inflamed, non-inflamed and control sites, at baseline, 15 days and 2 months. Kruskal-Wallis test and ANOVA test. *: difference between inflamed sites at baseline and 2 months; €: difference between inflamed sites at 15 days and 2 months; a: difference between inflamed and non-inflamed sites at 15 days; b: difference between inflamed and control sites; c: difference between non-inflamed and control sites; VEGF: Vascular endothelial growth factor; IL-10: Interleukin-10; MMP-8: Matrix metalloproteinase-8

Eighty-one samples were included for the gingival crevicular fluid analyses. IL-10, VEGF and MMP-8 were detected in gingival crevicular fluid collected at baseline, 15 days and 2 months (Figure 2). Our data showed a higher total amount of VEGF in inflamed sites in comparison to non-inflamed sites at all times. There were no differences between baseline and 2 months in all sites.

The total amount of IL-10 was higher in inflamed sites in comparison to non-inflamed sites at all times (p<0.05). Also, non-inflamed sites showed higher amounts of IL-10 in comparison to control sites at all times (p<0.05).

The total amount of MMP-8 had a reduction 15 days after periodontal therapy, but not statistically significant, and the total amount was higher in inflamed sites after two months (p<0.05). Also, it was higher in control sites in comparison to non-inflamed sites after 15 days (p<0.05).

### mRNA expression

**Figure 3:**
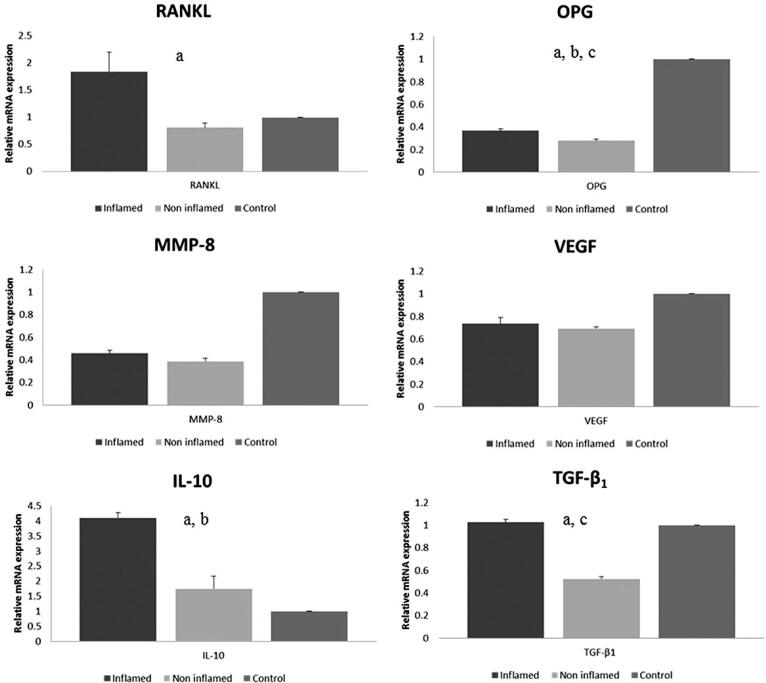
Relative mRNA expression of RANKL, OPG, MMP-8, VEGF, IL-10 and TGF-β1 in inflamed, non-inflamed and control sites. Kruskal-Wallis test and ANOVA test. a: significant difference between inflamed and non-inflamed sites b: significant difference between inflamed and control sites c: significant difference between control and non-inflamed sites. RANKL: Receptor activator of nuclear factor ĸB; OPG: Osteoprotegerin; VEGF: Vascular endothelial growth factor; IL-10: Interleukin-10; MMP-8: Matrix metalloproteinase-8; TGF-β1: Transforming growth factor- β1

We examined the expression of IL-10, RANKL, OPG, MMP-8, VEGF, and TGF-β_1_ in inflamed, non-inflamed and control sites after periodontal therapy. Comparisons between inflamed sites and non-inflamed sites, showed increased expression of IL-10 (p=0.03), RANKL (p<0.001) OPG (p=0.02), and TGF-β_1_ (p<0.05) in inflamed sites. Control sites demonstrated higher expression of OPG (p<0.001) and TGF-β_1_ (p<0.05) when compared to non-inflamed sites. Inflamed sites had higher expression of IL-10 when compared to control sites (p=0.026). MMP-8 and VEGF showed no differences (Figure 3).

## Discussion

In the present study, we monitored inflammation and progressive periodontal sites to investigate potential differences in the molecular profile of gingival crevicular fluid and gingival biopsies from inflamed and non-inflamed sites. Groups and sites were categorized in order to express significant clinical differences measured by periodontal parameters (PD a rCAL) and inflammation (BOP). Additionally, early changes in the clinical attachment levels were used to investigate the role of inflammatory markers in disease modulation.

Samples were collected at baseline, 15 days and 2 months after basic periodontal therapy. As expected, our data showed significant difference in clinical parameters between periodontitis group and control group at baseline. After periodontal therapy, data showed significant improvements on clinical parameters in periodontitis group. It was observed reduction in PD (0.7 mm ±0.4), BOP (37.1%±5.0), PI (27.2%±7.3), and rCAL gain (0.9 mm ±0.5). These results confirm the short-term beneficial effect of the therapy and are in accordance with previous data that showed better clinical conditions after full mouth disinfection^24^^,^ ^25^ or scaling and root planning over a 3- to 4- week period.^26^^,^ ^27^

Inflamed sites showed higher amount of IL-10 (0.29 pg ±0.09) in comparison to control sites (0.21 pg ±0.08) before treatment (p<0.05). Furthermore, IL-10 mRNA expression was higher in inflamed sites in comparison to non-inflamed and control sites. This is in accordance to some previous results.^28^^–^^30^ Goutoudi, et al.^31^ (2004) using a different methodology observed a similar amount of IL-10 when compared diseased and non-diseased sites instead of the inflamed sites classification of our study.

Periodontal disease activity is accepted as bone and attachment loss^32^ related to variations in inflammatory cells, migration of monocytes/macrophage^33^ and has been associated to inflammatory biomarkers.^7^^,^ ^34^^,^ ^35^ Our results found that 17% of total sites could be classified as progressive, according to the tolerance method.^20^^,^ ^21^^,^ ^36^ However, we did not find differences in the protein levels of MMP-8, VEGF and IL-10 in gingival crevicular fluid of progressive sites compared to inactive sites after therapy. Indeed, no association was observed between bleeding on probe and disease progression. A previous study observed a relationship between bleeding on probe and disease activity, but it is yet controversial and other authors showed similar results to ours.^37^

Interestingly, the higher expression of MMP-8 in inflamed sites observed in our study may explain why progressive sites also demonstrated higher IL-10 levels. The anti-inflammatory effect of IL-10 decreases the expression of pro-inflammatory cytokines, like TNF-alfa, IL-1β and matrix metalloproteinases (MMPs). Because of its protective function against bone loss, IL-10 inhibits MMPs^29^ through the up-regulation of Tissue Inhibitor of Metalloproteinase (TIMPs) that are capable of inhibiting almost every member of the MMP family^38^ Thus, the higher expression of IL-10 in inflamed sites may have moderated the destructive effect of Th1 response and may have been accounted for lowering the expression of MMP-8.^28^ Although clinical results demonstrated periodontal pocket reduction after periodontal therapy, some sites remained with probing depth >4 mm. This can explain the increase in MMP8 levels in 2 months, although its reduction after 15 days. Remaining periodontal pocket could increase inflammatory cytokines.

Furthermore, our site-specific analysis presented higher expression of RANKL mRNA in inflamed sites compared to non-inflamed sites. Inflamed sites also had higher expression of OPG mRNA compared to non-inflamed sites and, consequently, relative ratio RANKL/OPG mRNA was higher. Garlet, et al.^32^ (2004) observed higher expression of RANKL mRNA in chronic periodontitis patients compared to healthy patients, as well as higher expression of IL-10 mRNA. The expression of OPG was also higher, but not significant. According to the authors, higher expression of OPG could control the alveolar bone loss driven by RANKL, attenuating the progression and severity of the disease. The expression of RANKL and MMPs may result in tissue destruction and disease progression, whereas the higher expression of TIMPs and OPG possibly induced by IL-10, could be responsible for the control of tissue destruction.^29^ Indeed, these results are in agreement to ours and suggest that, in higher amounts, IL-10 could control bone resorption and moderate periodontal destruction.

We also found higher expression of TGF-β_1_ mRNA in inflamed sites compared to non-inflamed sites (p<0.05). Dutzan, et al.^41^ (2012) observed higher expression of TGF-β_1_ in healthy sites compared to periodontitis sites, which in our study showed no difference. Unlikely, we found higher expression of TGF-β_1_ mRNA in inflamed and control sites compared to non-inflamed sites, probably indicating the anti-inflammatory characteristic and modulatory role of TGF-β_1_ in inflamed sites, possibly promoting modulation of pro-inflammatory cytokines and stimulating the production of IL-1 receptor antagonist, which regulates anti-inflammatory and immunesupressor activities.^39^

Regarding VEGF, we found significant difference between inflamed sites and control sites at all times. This result is subject to bias given gingival tissue samples collected from sites that received periodontal therapy. Besides, the presence of VEGF in gingival fluid of healthy patients may reflect sub-clinical levels of inflammation, healing following bacterial assault or physiological angiogenesis in periodontal tissues.^40^

Despite having some sites with periodontal disease progression, our site-specific analysis also showed considerable levels of anti-inflammatory markers, possibly reducing risk for more attachment loss.

In conclusion, in spite of data analysis limitations and the short follow-up period to appreciate major disease breakdown, this preliminary study stressed out that progressive disease activity is a possible occurrence even after basic periodontal therapy, but is limited to a small percentage of sites. Also, periodontal treatment reduces elevated inflammation markers of inflamed sites from disease patients to levels of those observed in healthy subjects, but these levels could not be sustained in case of residual periodontal pockets. However, as elevated gene and protein anti-inflammatory marker levels in inflamed sites prior treatment could suggest its modulatory role, it does not seem to discriminate future progressive sites. Predictors of future attachment loss are still a challenge in periodontal diagnosis.
